# Integrating single-cell and spatial multi-omics for precision oncology: from tumor ecosystems to clinical decision-making

**DOI:** 10.3389/fonc.2026.1936192

**Published:** 2026-09-14

**Authors:** Sowhanur Rahman Nirob, Priya Rani Das, Md. Owafeeuzzaman Patwary, Syeda Aria Ashfaque, Md. Reazul Islam, Md. Shabiul Islam, Nibras Ahmed, Firoz Ahmed

**Affiliations:** 1 Department of Computer Science, American International University-Bangladesh (AIUB), Dhaka, Bangladesh; 2 Department of Computer Science, University at Albany, State University of New York (SUNY), Albany, NY, United States; 3 Centre for Advanced Devices and Systems, Centre of Excellence for Robotics and Sensing Technologies, Multimedia University, Persiaran Multimedia, Cyberjaya, Selangor, Malaysia; 4 Faculty of Artificial Intelligence and Engineering, Multimedia University, Persiaran Multimedia, Cyberjaya, Selangor, Malaysia; 5 Department of Computer Science, Rajshahi University of Science and Technology, Rajshahi, Bangladesh

**Keywords:** biomarker discovery, clinical translation, precision oncology, single-cell sequencing, spatial multi-omics, tumor heterogeneity, tumor microenvironment

## Abstract

The integration of single-cell and spatial multi-omics technologies has substantially advanced our understanding of cancer as a dynamic ecosystem characterized by profound intratumoral heterogeneity and complex microenvironmental interactions. While traditional bulk sequencing approaches average signals across millions of cells, obscuring critical diversity that drives therapeutic resistance, these advanced platforms may enable simultaneous profiling of genomic, transcriptomic, epigenetic, and proteomic information while preserving spatial architecture. This mini-review synthesizes recent technological advances and clinical applications of integrated single-cell and spatial multi-omics for precision oncology, employing a structured narrative literature search across primary databases spanning 2021 to 2026. The convergence of these technologies may enable comprehensive tumor ecosystem mapping, may facilitate spatially informed biomarker discovery for patient stratification, has shown potential to predict immunotherapy response, and may guide rational combination therapy selection. Artificial intelligence holds promise as a computational engine for harmonizing heterogeneous datasets, with the potential to support future real-time clinical decision-making and adaptive treatment strategies. The proposed conceptual workflow provides a stepwise translational roadmap from multi-omics discovery to clinical implementation. However, it is important to note that many of these applications remain at an early stage, with most evidence derived from small cohorts, preclinical models, or retrospective analyses, and prospective clinical validation is still needed. Despite significant barriers including technical standardization, computational complexity, high costs, and regulatory challenges, the integration of single-cell and spatial multi-omics holds immense promise for advancing personalized precision oncology, with the ultimate goal of improving patient outcomes through dynamic, response-adaptive therapeutic approaches.

## Highlights

- To provide a critical synthesis of recent technological breakthroughs in single-cell and spatial multiomics, highlighting their individual strengths and synergistic potential for comprehensive tumor characterization.
- To offer a comprehensive assessment of the major translational barriers, including technical, computational, and regulatory challenges that currently impede the routine clinical adoption of these advanced omics platforms.
- To propose a novel, unified conceptual workflow that integrates single-cell genomics with spatial multi-omics for systematic tumor ecosystem mapping, enabling the discovery of spatially informed biomarkers, guiding patient stratification, directly informing clinical decision-making, and ultimately advancing the practice of precision oncology.
- While these contributions reflect the current trajectory of the field, it is important to recognize that many of the proposed applications remain in early stages of development. Most evidence cited in this review derives from small cohort studies, preclinical models, or retrospective analyses, and prospective clinical validation is required before these approaches can be integrated into routine oncology practice.

## 1 Introduction

Cancer functions as a dynamic and evolving ecosystem, wherein malignant cells coexist with immune infiltrates, stromal fibroblasts, and vascular networks, collectively dictating disease progression and therapeutic response. Single-cell genomics has fundamentally transformed our ability to dissect tumor heterogeneity by revealing the genetic, transcriptional, and epigenetic diversity of individual cancer cells (1, 2). However, this high-resolution cellular profiling is typically performed on dissociated tissues, thereby losing the spatial tissue architecture that is critical for understanding cell–cell communication and microenvironmental niches. Spatial multi-omics technologies, including spatial transcriptomics and spatial proteomics, have emerged to complement this gap, enabling the mapping of tumor, immune, and stromal cell interactions directly within their native spatial context (3, 4). The convergence of these two technological pillars, single-cell and spatial multi-omics, offers a promising opportunity to construct a comprehensive atlas of the tumor ecosystem. The scope of this mini-review is to critically synthesize recent advances in this integrative field and to outline a potential translational pathway that leverages these multimodal data to improve patient stratification, guide combination therapy selection, and enable real-time treatment monitoring in precision oncology.

Despite the rapid proliferation of these technologies, existing reviews and current research suffer from several critical limitations that hinder clinical translation. First, many reviews focus exclusively on single-cell genomics for characterizing tumor heterogeneity but provide limited discussion of spatial tissue architecture, thereby overlooking the structural organization that governs cellular communication (5). Conversely, reviews dedicated to spatial transcriptomics or spatial omics often treat these as standalone technologies and lack integration with high-resolution single-cell molecular profiling, leading to an incomplete picture of the tumor microenvironment. Second, a substantial body of literature concentrates on technology development and sequencing platforms, yet places limited emphasis on clinical translation, leaving a significant gap between bench-side discovery and bedside application (6). Third, discussions of tumor heterogeneity are predominantly framed from a genomic perspective, while spatial cell–cell interactions and the functional states of immune and stromal populations are frequently underrepresented. Fourth, conventional biomarker discovery efforts rely heavily on bulk genomic approaches, and few reviews focus on identifying clinically actionable spatial biomarkers that can inform prognosis or predict therapeutic sensitivity. Fifth, artificial intelligence and multi-omics integration are typically reviewed in isolation, with limited integration into spatial biology and practical clinical workflows (7). Sixth, even when omics applications are described, there is insufficient critical evaluation of barriers to routine clinical implementation, including issues of standardization, reproducibility, scalability, and regulatory compliance (8, 9). Finally, most broad overviews of precision oncology lack a unified conceptual framework that systematically connects molecular profiling to clinical decision-making (10). These collective gaps underscore the urgent need for a cohesive, clinically-oriented synthesis.

To address these limitations, a synergistic integration of single-cell genomics and spatial multi-omics is proposed to provide a comprehensive, multidimensional view of the tumor ecosystem. The novelty of this review lies in presenting a unified conceptual framework that explicitly bridges high-resolution molecular profiling with actionable clinical outcomes. The true power of these technologies emerges not from their independent application, but from their combination to map tumor, immune, and stromal cell interactions within their precise spatial coordinates. This integrated approach may enable the discovery of spatially informed biomarkers that could be superior to conventional genomic markers for patient stratification, prognosis, and treatment selection. Importantly, the role of artificial intelligence as a potential computational engine to harmonize these massive, heterogeneous datasets is discussed, potentially enabling integration into precision oncology workflows. Critically, this review goes beyond descriptive discovery by outlining a translational pipeline that addresses the barriers to routine clinical implementation, including standardization of protocols, reproducibility of spatial assays, scalability for large-scale studies, and strategies for regulatory approval. The proposed conceptual workflow follows a stepwise progression from single-cell genomics and spatial multi-omics to tumor ecosystem mapping, spatial biomarker discovery, patient stratification, clinical decision-making, and ultimately the delivery of precision oncology.

## 2 Methodology of literature selection

This mini-review followed a structured narrative literature search guided by the PRISMA 2020 reporting framework to ensure a transparent and reproducible study selection process. Literature was retrieved from five major scientific databases: PubMed, SpringerLink, PubMed Central (PMC), ScienceDirect, and Wiley Online Library. The final search was conducted on March 15, 2026. Publications from January 2021 to March 2026 were considered to capture recent advances in single-cell and spatial multi-omics technologies for precision oncology.

The search strategy combined controlled vocabulary and free-text keywords using Boolean operators (AND, OR). Core search terms included *“single-cell sequencing,” “single-cell multi-omics,” “spatial transcriptomics,” “spatial omics,” “tumor heterogeneity,” “tumor microenvironment,” “precision oncology,” “cancer biomarkers,” “artificial intelligence,” “machine learning,” “multi-omics integration,”* and *“clinical translation.”* Representative database-specific search strings were adapted according to each platform’s indexing system. In addition, the reference lists of relevant review articles were manually screened to identify eligible studies not captured through the electronic database search.

The initial search identified 120 records. Duplicates removed 22, records after duplicates removed 98, which remained for title and abstract screening. Full-text assessment was subsequently performed for 50 articles. Following detailed eligibility evaluation, 25 studies satisfied all predefined inclusion criteria and were included in this mini-review.

Title/abstract screening and full-text eligibility assessment were conducted independently by three reviewers. Any disagreements were resolved through discussion and consensus. When consensus could not be reached, final decisions were made by the senior authors according to the predefined eligibility criteria.

Eligible studies were evaluated for methodological quality, scientific relevance, and clinical significance using a structured qualitative assessment framework adapted for narrative review synthesis. Methodological quality was assessed based on sample size adequacy, use of orthogonal validation techniques, technical replicate consistency, and adherence to established experimental guidelines. Scientific relevance was evaluated according to conceptual novelty, hypothesis-driven experimental design, and contribution to mechanistic understanding of tumor biology. Clinical significance was determined by the presence of direct patient-derived data, correlation with therapeutic outcomes, potential for actionable biomarker strategies, and translational feasibility. Based on these criteria, studies were assigned a priority score (high, moderate, or low) through independent evaluation by three reviewers, with discrepancies resolved via discussion and senior author adjudication. Studies categorized as high priority provided robust multi-modal integration with direct clinical implications and were weighted more heavily in the thematic synthesis. Particular emphasis was placed on studies investigating tumor heterogeneity, tumor microenvironment characterization, biomarker discovery, patient stratification, therapeutic response prediction, and computational integration of single-cell and spatial multi-omics data. Studies focused solely on methodological development without clinical application, bulk sequencing approaches, or non-oncology topics were excluded. The complete literature selection workflow is illustrated in **Figure 1**.

**Figure 1:**
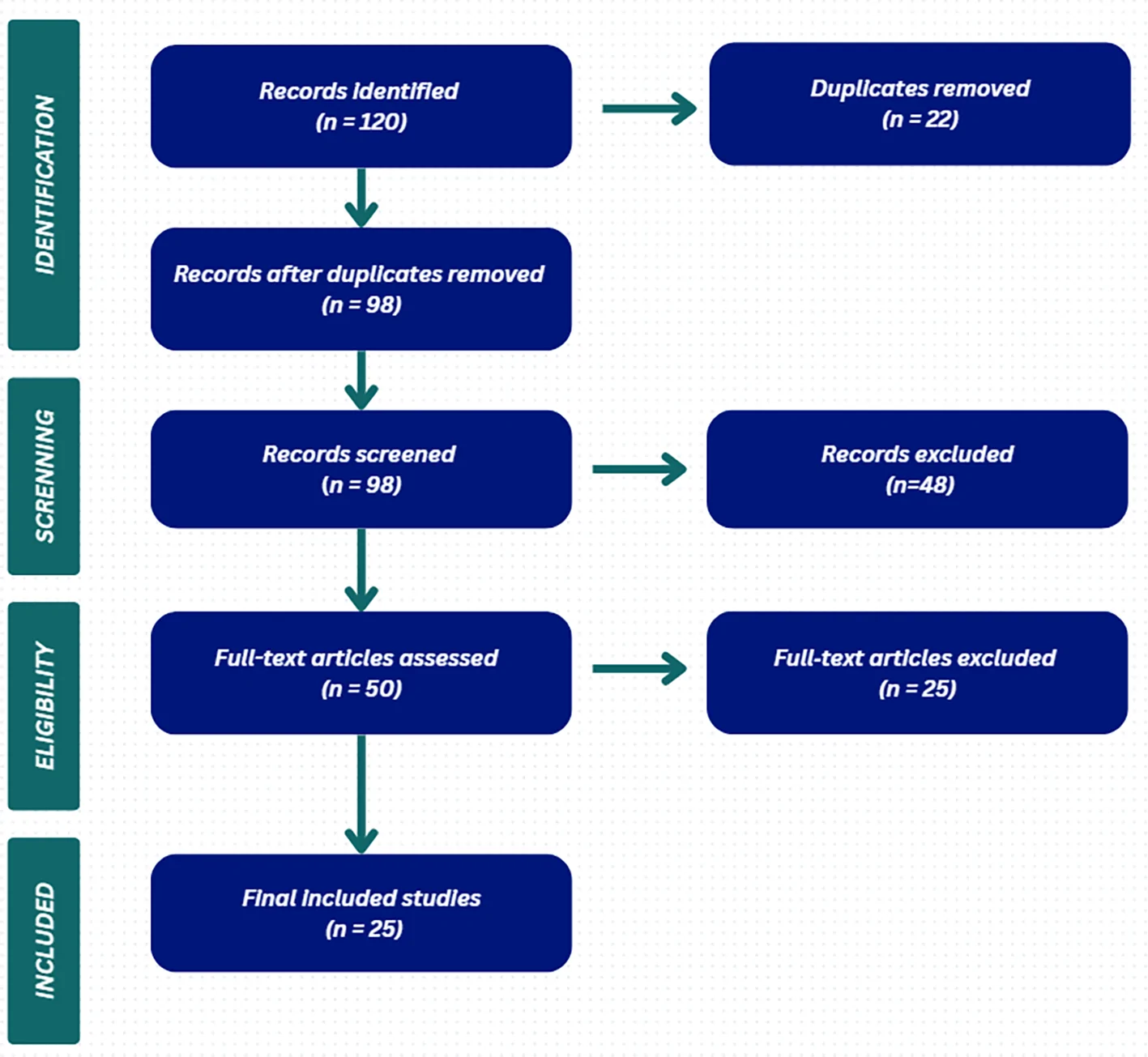
PRISMA 2020 flow diagram illustrating the literature identification, screening, eligibility assessment, and final study selection process.

### 2.1 Inclusion criteria

- Articles published between January 2021 and March 2026.
- Peer-reviewed original research articles, systematic reviews, and high-quality review papers.
- Studies investigating single-cell omics technologies (e.g., scRNA-seq, scATAC-seq, scDNA-seq, single-cell proteomics, or multi-omics).
- Studies involving spatial omics technologies, including spatial transcriptomics, spatial proteomics, or spatial epigenomics.
- Research integrating single-cell and spatial omics for cancer biology or precision oncology.
- Studies addressing tumor heterogeneity, tumor microenvironment, biomarker discovery, or patient stratification.
- Studies employing computational approaches such as artificial intelligence, machine learning, or multi-omics data integration.
- Studies with demonstrated clinical or translational relevance.
- Articles published in English.

### 2.2 Exclusion criteria

- Articles published before January 2021.
- Studies unrelated to cancer or precision oncology.
- Research based exclusively on bulk sequencing without single-cell or spatial resolution.
- Studies describing only technical methodology without biological or clinical application.
- Conference abstracts, editorials, letters, perspectives, commentaries, and opinion articles.
- Duplicate publications or substantially overlapping reports.
- Studies with insufficient methodological detail or poor scientific quality.
- Non-English publications.

Although this review is specifically focused on the integration of single-cell and spatial multi-omics, the included studies varied considerably in their degree of technological integration and clinical applicability. To ensure transparent synthesis, the 25 included studies were categorized into four evidence tiers based on the level of integration and translational relevance:

- Tier 1 – Fully Integrated Single-Cell and Spatial Multi-Omics (n = 8): Studies that simultaneously employed single-cell and spatial multi-omics technologies within the same cohort, directly integrating molecular profiles with spatial architecture to address clinically relevant questions.
- Tier 2 – Spatial Multi-Omics with Single-Cell Integration (n = 6): Studies primarily utilizing spatial transcriptomics, spatial proteomics, or true spatial multi-omics while incorporating single-cell reference datasets or deconvolution approaches to enhance cellular resolution.
- Tier 3 – Single-Cell Multi-Omics with Spatial Interpretation (n = 7): Studies employing singlecell multi-omics that, although lacking direct spatial measurements, provided substantial contextual analysis of spatial tissue architecture, cell–cell interactions, or microenvironmental organization with clear translational implications.
- Tier 4 – Supportive Computational and Translational Studies (n = 4): Studies focusing on computational integration frameworks, artificial intelligence pipelines, or clinical implementation strategies that, although not generating primary experimental data, provided essential methodological or translational guidance for integrating these technologies into clinical settings.

Throughout the narrative synthesis, evidence from the higher tiers (Tiers 1–2) was prioritized for claims regarding direct technological integration and spatial biology, whereas Tiers 3–4 were used to support contextual discussions of single-cell resolution, computational harmonization, and clinical translation pathways. This tiered categorization ensures that the review’s conclusions are appropriately grounded in studies providing the strongest evidence of true single-cell and spatial multi-omics integration, while also capturing the breadth of relevant translational research in this rapidly evolving field.

## 3 Technological advances in single-cell and spatial multi-omics

The rapid evolution of single-cell and spatial multi-omics technologies has substantially advanced the landscape of cancer research, enabling unprecedented resolution in dissecting tumor biology. Single-cell sequencing platforms have advanced from early transcriptomic approaches to comprehensive multi-omic profiling that captures genomic, epigenomic, and proteomic information simultaneously from individual cells. These technological innovations have been instrumental in revealing the extent of intratumoral heterogeneity and identifying rare subpopulations that drive therapy resistance and disease progression (11). Concurrently, spatial multi-omics technologies, including spatial transcriptomics and spatial proteomics, have emerged to address the critical limitation of single-cell approaches, namely the loss of spatial context during tissue dissociation. Spatial transcriptomics and spatial proteomics platforms now enable the mapping of molecular signatures directly onto tissue architecture, preserving the spatial coordinates of cells within their native microenvironment (12).

A critical evaluation of available platforms reveals fundamental trade-offs that must be carefully considered for clinical translation (4). Single-cell platforms vary primarily in throughput, transcript coverage, and cost. Droplet-based systems such as 10x Genomics Chromium offer high throughput (80,000 cells/run) with robust reproducibility but are limited to 3’ transcript counting, whereas plate-based methods like Smart-seq2 provide full-length coverage at the cost of lower throughput (96–384 cells/plate). For single-cell multi-omic applications, platforms such as 10x Multiome enable simultaneous profiling of mRNA and chromatin accessibility from the same cell, though at substantially higher cost and computational complexity.

Spatial transcriptomics platforms exhibit even more pronounced trade-offs between resolution, gene coverage, and sample compatibility. Sequencing-based approaches such as 10x Visium (55 µm spots) and Visium HD (2 µm) provide unbiased whole-transcriptome coverage but at moderate to high resolution. In contrast, BGI Stereo-seq achieves subcellular resolution (0.5 µm spots) with large-area coverage, though at the cost of enormous data volume and lower per-spot sensitivity. Imaging-based platforms including MERFISH, Xenium, and CosMx offer true single-cell to subcellular resolution but are restricted to targeted gene panels (300–6,000 genes), limiting discovery potential. Importantly, FFPE compatibility critical for clinical archival samples varies widely, with Visium HD and CosMx SMI offering robust FFPE workflows, whereas Stereo-seq and Slide-seq remain largely limited to fresh-frozen tissues. True spatial multi-omics, which simultaneously profiles multiple molecular layers (e.g., transcriptomics and proteomics) from the same tissue section, remains an emerging area with platforms such as CosMx SMI and GeoMx DSP leading this integration.

A significant technological breakthrough has been the development of long-read sequencing platforms for cancer liquid biopsy applications. These technologies overcome the limitations of short-read sequencing by enabling comprehensive characterization of structural variants, fusion genes, and epigenetic modifications from circulating tumor DNA and other liquid biopsy analytes. The integration of long-read sequencing with single-cell approaches offers enhanced resolution for detecting complex genomic rearrangements and monitoring tumor evolution through non-invasive means, thereby complementing the spatial resolution provided by spatial multi-omics technologies (13). Furthermore, advances in microfluidics have revolutionized single-cell isolation and processing, enabling high-throughput analysis with reduced costs and improved reproducibility. These platforms facilitate the capture of rare circulating tumor cells and the precise manipulation of single cells for downstream single-cell multi-omic analysis, providing the cellular resolution needed to interpret spatial transcriptomic data (11).

The computational landscape has evolved in parallel with experimental technologies to address the formidable challenge of integrating massive, heterogeneous datasets generated by single-cell and spatial multi-omics platforms. Artificial intelligence and machine learning algorithms have become indispensable tools for harmonizing multi-modal data, identifying hidden patterns, and extracting biologically meaningful insights from complex molecular landscapes. These computational approaches enable the integration of transcriptomic, epigenomic, and proteomic data layers, facilitating the reconstruction of cellular states, trajectories, and regulatory networks (14). The development of sophisticated data integration frameworks has been particularly crucial for combining single-cell and spatial data, enabling the projection of high-resolution molecular profiles onto spatial coordinates and the inference of cell–cell communication networks within the tumor microenvironment (15).

Looking forward, emerging technologies may further enhance the resolution and clinical applicability of single-cell and spatial multi-omics approaches. The convergence of these platforms with artificial intelligence-driven biomimetic nanoplatforms represents a frontier in precision oncology, potentially enabling intelligent and precise intervention in the immunosuppressive core regions of tumors (15). As these technologies continue to mature, their translation from research laboratories to clinical settings will depend on addressing challenges related to standardization, reproducibility, and costeffectiveness (14). **Table 1** presents key technological platforms for single-cell and spatial multi-omics integration. **Table 2** presents clinical applications, translational impact, and implementation barriers of integrated single-cell and spatial multi-omics. **Table 3** provides a comparative summary of key platforms across clinically and experimentally relevant dimensions, facilitating informed platform selection for translational research.

**Table 1:** Technological advances in single-cell and spatial multi-omics for precision oncology.

Citation	Technology	Key advances	Challenges & limitations
Guzman and Rodriguez (13)	Long-read sequencing	Comprehensive characterization of structural variants, fusion genes, and epigenetic modifications from ctDNA; overcomes short-read limitations; enables real-time clonal tracking	High cost; complex bioinformatics; limited clinical adoption; validation required
Cen et al. (9)	Single-cell spatial multi-omics	scRNA-seq, scATAC-seq, spatial transcriptomics methods; enables multi-modal molecular profiling while preserving spatial architecture	Technical variability; batch effects; low capture efficiency; high cost per sample; standardization lacking
Inayatullah et al. (1)	Single-cell omics	Transformative clinical applications; biomarker discovery; technological evolution from transcriptomics to comprehensive multi-omic profiling	Complex data analysis; integration with clinical workflows; standardization; high cost; limited accessibility
Pan and Jia (2)	Single-cell multiomics	Dissects cancer cell plasticity and tumor heterogeneity; reveals therapy-resistant clones and stemlike properties	Limited spatial context; loss of tissue architecture; data integration challenges; high computational demands
Kim and Takahashi (11)	Emerging singlecell technologies	Multi-omic integration; highresolution molecular profiling; rare cell identification; drug development applications	Integration challenges; high cost; standardization; computational demands; limited clinical validation
Li et al. (6)	Bulk single-cell multi-omics	Systems biology approaches; precision medicine applications; integrative analysis of multi-modal data	Data heterogeneity; integration challenges; computational complexity; standardization issues
Wu et al. (7)	Single-cell to multi-omics	Comprehensive technologies and applications; data analysis pipelines; clinical integration roadmap	Complex integration; standardization challenges; data formats; high cost; limited clinical validation
Lim et al. (4)	Single-cell multiomics	High-resolution profiling; computational approaches; drug development; rare cell identification	High computation; data storage; integration challenges; batch effects; standardization; high cost
Ma et al. (12)	Single-cell multiomics	Drug discovery and development; clinical translation; biomarker identification; precision pharmacology	Complex analysis; high cost; standardization; validation need; regulatory hurdles; integration challenges
Carneiro et al. (8)	Microfluidics	Single-cell isolation; highthroughput analysis; CTC capture; liquid biopsy integration; minimal sample requirements	Technical variability; high cost; limited multiplexing; standardization; clinical validation needed
Huang et al. (16)	Single-cell multi-omics + ML	Machine learning integration; stemness signature identification; computational modeling; therapeutic targeting	Complex pipelines; explainable AI need; clinical validation; high computation; integration challenges
Liu et al. (14)	AI for multi-omics	Machine learning harmonization; heterogeneous data integration; clinical translation; decision support	Explainable AI need; clinical validation; high computation; privacy concerns; regulatory challenges

**Table 2:** Clinical applications, translational impact, and implementation barriers of integrated single-cell and spatial multi-omics.

Citation	Application area	Clinical impact & translation	Challenges & barriers
Qureshi et al. (5)	Liquid biopsy/ Early detection	Non-invasive early cancer detection; real-time treatment monitoring; minimal residual disease detection; adaptive therapy guidance	Low ctDNA detection at low fractions; standardization lacking; high cost; ethical concerns; prospective validation needed
Li et al. (17)	Spatial biomarker discovery	Identifies immune low-response states; predicts immunotherapy response; enables patient stratification; guides combination therapy	Limited to endometrial cancer; small cohort; reproducibility issues; high cost; validation across platforms needed
Barjij et al. (18)	Patient stratification	Systematic characterization of spatial & temporal heterogeneity; predicts treatment response; guides adaptive treatment strategies	Limited to breast cancer; study heterogeneity; biomarker standardization; prospective validation required
Tang et al. (10)	Precision oncology platforms	Genomic profiling for therapy selection; combination therapy design; real-time monitoring; personalized treatment planning	Limited actionable biomarkers; high cost; standardization issues; reimbursement challenges; clinical validation needed
Du et al. (3)	Spatial multi-omics/TME mapping	Identifies spatially resolved biomarkers; predicts immunotherapy response; characterizes tumor microenvironment heterogeneity; guides targeted therapy	High cost; limited multiplexing; image analysis standardization; integration challenges; clinical validation needed
Le et al. (19)	Immunotherapy response	Reveals immune evasion mechanisms; predicts immunotherapy response; supports combination strategies; enables personalized immunotherapy selection	Limited spatial context; high cost; complex analysis; clinical cohort validation; integration with clinical workflows
Veo et al. (20)	Therapy resistance	Identifies metabolism-epigenetic reprogramming; reveals novel therapeutic vulnerabilities; guides combination therapy design	Limited to medulloblastoma; small cohort; high cost; complex experimental design; validation in larger cohorts
Nomura et al. (21)	Disease monitoring	Longitudinal methylation profiling; identifies epigenetic biomarkers; guides therapy timing; enables treatment adaptation	Limited to IDH-mutant glioma; high cost; tissue biopsy required; prospective validation needed
Tang et al. (22)	TME characterization	Decodes heterogeneity and microenvironment; predicts treatment response; guides combination strategies for biliary tract cancers	Limited to biliary tract cancers; small cohort; reproducibility; high cost; validation across institutions needed
He et al. (23)	TME-responsive therapy	Tumor microenvironment monitoring; precision nanomedicine; smart drug delivery; TME modulation; combination therapy design	Limited clinical validation; complex manufacturing; regulatory hurdles; high cost; translation barriers
Weng et al. (24)	Tumor evolution	Recurrent intra-tumour heterogeneity; metastatic prostate cancer characterization; therapy resistance understanding	Limited to prostate cancer; small cohort; high cost; reproducibility; validation in larger cohorts; complex analysis
Li et al. (15)	AI-guided nanoplatforms	Biomimetic nanoplatform design; AI-guided precision therapy; immunosuppressive TME targeting; real-time intervention	Preclinical stage; limited clinical validation; complex manufacturing; regulatory hurdles; high cost; translation barriers

**Table 3:** Comparative analysis of single-cell and spatial multi-omics platforms.

Platform	Type	Spatial resolution	Throughput	Molecular coverage	Sample compatibility	Key strengths/limitations
10x Genomics Chromium	scRNA-seq	Single-cell (droplet)	~10,000 cells/channel; ~80,000 cells/run	3*’* transcriptome (~500–1,500 genes/cell)	Fresh, frozen, FFPE (FLEX)	*Strengths:* Widely adopted, reproducible, and scalable. *Limitations:* 3*’* bias and limited full-length transcript coverage.
Smart-seq2/3	scRNA-seq	Single-cell (FACS/plate)	96–384 cells/plate	Full-length transcriptome	Pre-isolated single cells	*Strengths:* High sensitivity and full-length transcript coverage. *Limitations:* Low throughput, higher per-cell cost, and amplification bias.
BD Rhapsody	scRNA-seq	Single-cell (microwell)	>220,000 cartridge	3*’* transcriptome	Fresh, frozen	*Strengths:* High viability tolerance, bead-based recovery, and multiplexing. *Limitations:* Complex workflow and lower throughput than some droplet platforms.
10x Genomics Multiome	Single-cell multi-omics	Single-cell	Up to 20,000 nuclei; 160K nuclei/chip	mRNA + chromatin accessibility	Fresh, frozen	*Strengths:* Joint transcriptomic and epigenomic profiling. *Limitations:* High cost and complex multi-omic data integration.
10x Visium	Spatial transcri−ptomics	~55 *μ*m spot (5–50 cells)	~5,000 spots/slide	Whole transcriptome	FF, FFPE (targeted)	*Strengths:* High throughput, broad gene coverage, and H&E compatibility. *Limitations:* Moderate spatial resolution and multiple cells per spot.
10x Visium HD	Spatial transcri−ptomics	~2 *μ*m tiles (subcellular, single-cell scale)	~11 million barcoded squares per capture area	Whole transcriptome	FF, FFPE	*Strengths:* Near single-cell resolution and FFPE compatibility. *Limitations:* High sequencing requirements, large data volumes, and specialized analysis.
BGI Stereo-seq	Spatial transcri−ptomics	0.5 *μ*m spots (subcellular)	cm-scale tissue area	Whole transcriptome	FF, frozen	*Strengths:* Ultra-high spatial resolution and large tissue-area coverage. *Limitations:* Very large datasets, lower per-spot sensitivity, and limited accessibility.
Slide-seq V2	Spatial transcri−ptomics	~10 *μ*m bead (near single-cell)	~20,000 beads	Whole transcriptome	FF	*Strengths:* High spatial resolution with genome-wide profiling. *Limitations:* Lower sensitivity and technically demanding bead preparation.
MERFISH	Imaging-based spatial	Single-cell to subcellular (0.1–1 *μ*m)	Up to ~10,000 genes	Targeted gene panels	FF, fixed	*Strengths:* True single-cell resolution and precise RNA localization. *Limitations:* Targeted gene panels, lengthy iterative imaging, and specialized equipment.
Nanostring CosMx SMI	Imaging-based spatial multi-omics	Single-cell and subcellular	Up to 6,000 genes (or 18,000 with WTx assay) + proteins	Targeted panels	FFPE, FF	*Strengths:* FFPE compatibility and RNA–protein co-detection (true spatial multi-omics). *Limitations:* Targeted gene coverage and specialized imaging requirements.
10x Xenium	Imaging-based spatial	Subcellular	Up to 5,000 genes	Targeted panels	FFPE, FF, frozen	*Strengths:* Subcellular resolution and compatibility with post-assay H&E/IHC. *Limitations:* Restricted to targeted gene panels and relatively high cost per sample.
Nanostring GeoMx DSP	ROI-based spatial multi-omics	ROI-defined (10–50 *μ*m)	Up to 96 proteins or whole transcriptome (RNA)	ROI-based	FFPE, FF	*Strengths:* Compatible with standard FFPE and enables tumor–stroma ROI selection; true spatial multi-omics capability. *Limitations:* Not inherently single-cell and dependent on ROI selection.

## 4 Tumor ecosystem mapping through integrated omics

The integration of single-cell and spatial multi-omics has substantially advanced the understanding of cancer as a complex ecosystem rather than a homogeneous mass of proliferating cells. This paradigm shift has enabled the comprehensive mapping of tumor architecture, revealing the intricate relationships between malignant cells and their surrounding microenvironment. The convergence of these technologies provides unprecedented resolution for dissecting the cellular and spatial heterogeneity that drives tumor progression, therapeutic resistance, and metastatic dissemination.

This framework represents a conceptual roadmap for integrating these technologies, recognizing that many of the proposed applications remain in early stages of development and require prospective clinical validation before routine implementation. The proposed framework (**Figure 2**) begins with comprehensive tumor ecosystem mapping, capturing intratumoral heterogeneity, microenvironmental architecture, immunestromal networks, and clonal evolution. This systematic characterization provides the biological foundation for subsequent biomarker discovery. Importantly, this framework is presented as a conceptual and prospective roadmap rather than an established clinical pipeline. The pathway from molecular profiling to clinical outcomes including personalized treatment selection, improved patient outcomes, reduced toxicity, and prolonged remission should be interpreted as a vision for the field, with each step requiring rigorous validation before clinical adoption.

**Figure 2:**
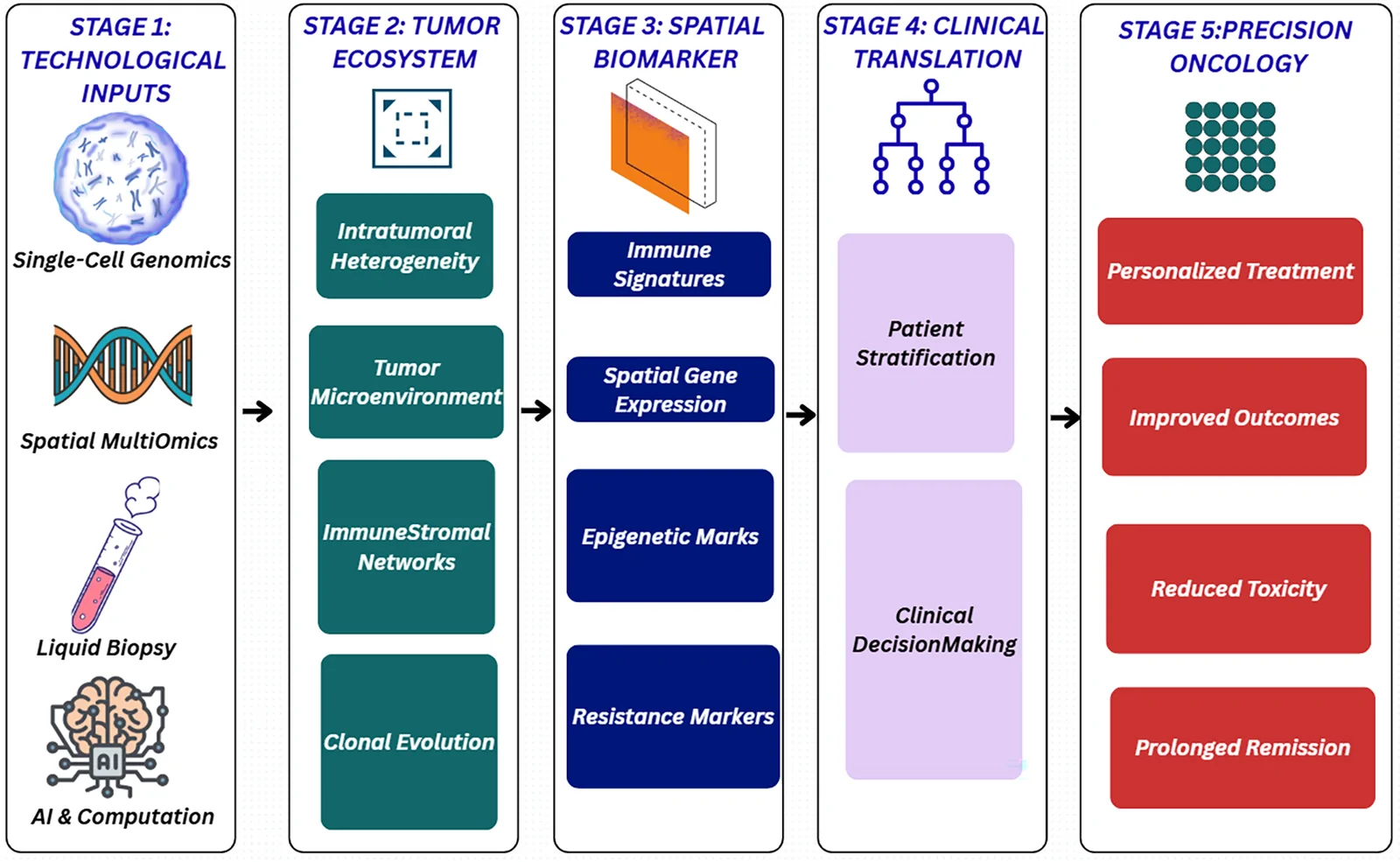
Conceptual framework for integrating single-cell and spatial multi-omics in precision oncology.

### 4.1 Decoding intratumoral heterogeneity at single-cell resolution

Intratumoral heterogeneity represents one of the most significant challenges in precision oncology, as diverse subpopulations within a single tumor exhibit distinct molecular profiles, proliferative capacities, and therapeutic vulnerabilities. Single-cell multi-omics has substantially advanced the characterization of this heterogeneity by enabling the simultaneous profiling of genomic, transcriptomic, and epigenomic features from individual cancer cells (2). This high-resolution approach has revealed the existence of rare subclones that possess stem-like properties and enhanced plasticity, enabling them to adapt to therapeutic pressures and drive disease progression. The identification of these therapy-resistant populations through single-cell analysis has provided valuable insights into the mechanisms underlying treatment failure and has guided the development of combination strategies designed to target multiple subclones simultaneously (20).

### 4.2 Mapping the tumor microenvironment and cell-cell interactions

Beyond characterizing cancer cells themselves, integrated single-cell and spatial multi-omics has provided unprecedented insights into the tumor microenvironment, encompassing immune infiltrates, stromal fibroblasts, endothelial cells, and the extracellular matrix. The spatial organization of these components within the tumor architecture plays a critical role in determining immune surveillance, therapeutic response, and clinical outcomes (19). Spatial transcriptomics and spatial proteomics have enabled the mapping of immune cell infiltration patterns, revealing the existence of immune-excluded, immuneinfiltrated, and immune-desert phenotypes that correlate with differential responses to immunotherapy. Furthermore, the identification of specific ligand-receptor interactions and paracrine signaling networks through spatial omics has provided insights into the mechanisms by which cancer cells remodel their microenvironment to evade immune destruction and promote tumor growth (22).

### 4.3 Temporal dynamics and clonal evolution

The temporal dimension of tumor evolution represents a critical aspect of cancer biology that has been illuminated through integrated multi-omics approaches. Longitudinal profiling of tumors through single-cell and spatial multi-omics has revealed the dynamic nature of clonal evolution, demonstrating how subclonal populations expand, contract, and acquire new mutations in response to therapeutic selection pressures (21). This temporal perspective has been particularly informative for understanding the emergence of therapy resistance, as repeated profiling of tumors during treatment has identified the sequential acquisition of resistance mutations and the switching of cellular states that enable survival under therapeutic stress. The integration of liquid biopsy approaches with single-cell and spatial multi-omics has further enhanced the ability to monitor tumor evolution non-invasively, potentially enabling the detection of emerging resistance clones and the adaptation of treatment strategies in real-time, though this remains an area of active investigation (20).

## 5 Clinical applications and translational pipeline

The translation of single-cell and spatial multi-omics from research laboratories to clinical practice represents the ultimate frontier in precision oncology. This translational pipeline encompasses the discovery of spatially informed biomarkers, integration with non-invasive monitoring approaches, harmonization of complex datasets through artificial intelligence, and the application of these insights to guide rational therapy selection and adaptive treatment strategies. While these applications show considerable promise, it is important to acknowledge that most evidence remains preliminary. Many studies are limited by small sample sizes, lack of prospective validation, and reliance on preclinical models. The following sections discuss these applications with appropriate caution, distinguishing between established clinical utility and emerging or potential applications.

### 5.1 Spatial biomarker discovery for patient stratification and prognosis

The integration of spatial omics with single-cell resolution has advanced biomarker discovery by revealing molecular signatures that appear intrinsically linked to tissue architecture and cellular organization. Spatial biomarkers, defined as molecular features whose predictive or prognostic value depends on their precise location within the tumor microenvironment, may offer complementary and often enhanced predictive value over conventional genomic markers (17). These spatially resolved biomarkers capture the heterogeneity of immune infiltration patterns, the organization of tertiary lymphoid structures, and the spatial relationships between cancer cells and stromal elements that collectively determine clinical outcomes. Preliminary studies have suggested that spatial immune exclusion signatures, characterized by the physical separation of cytotoxic T cells from tumor cells, may serve as promising predictors of immunotherapy resistance, with early evidence indicating potential for improved patient stratification compared to traditional histopathological assessment alone (18). However, these findings require validation in larger, prospective cohorts before clinical implementation (3).

### 5.2 Integration with liquid biopsy for non-invasive monitoring

The convergence of spatial omics with liquid biopsy technologies presents a promising opportunity for longitudinal, non-invasive monitoring of tumor evolution. Single-cell and spatial analyses provide the molecular blueprint of tumor heterogeneity, which can be leveraged to design sensitive liquid biopsy assays that capture the full spectrum of circulating tumor-derived analytes, including circulating tumor DNA, circulating tumor cells, and tumor-educated platelets (25). This integrated approach, which combines the cellular resolution of single-cell profiling with the spatial context provided by spatial multiomics, may enable real-time tracking of clonal dynamics, early detection of emerging resistance mutations, and monitoring of minimal residual disease following therapy (5). The combination of high-resolution spatial maps with minimally invasive liquid biopsies offers a potentially powerful strategy for personalized treatment adaptation, allowing clinicians to detect therapeutic failure earlier and switch to alternative regimens before clinical progression becomes evident (13). Prospective studies are needed to validate the clinical utility of this approach.

### 5.3 Artificial intelligence and machine learning for multi-omics data integration

The effective integration of single-cell and spatial multi-omics datasets represents a formidable computational challenge that is being addressed through artificial intelligence and machine learning approaches. These computational frameworks facilitate the harmonization of heterogeneous data modalities, assist in the identification of latent biological patterns, and support the extraction of clinically actionable insights from complex molecular landscapes (14). Machine learning algorithms, particularly deep learning architectures, have shown substantial capability in preliminary studies for integrating transcriptomic, epigenomic, and proteomic data layers to reconstruct cellular states, identify patterns suggestive of regulatory networks, and generate predictions of therapeutic responses (7). The development of explainable artificial intelligence models is particularly critical for clinical translation, as interpretable predictions allow clinicians to better interpret the biological basis of algorithmic recommendations and build trust in computational decision support tools (16). However, rigorous prospective validation in diverse patient populations is required before these models can be deployed in clinical settings.

### 5.4 Guiding combination therapy selection and adaptive treatment strategies

By revealing the specific vulnerabilities of distinct tumor subpopulations and the spatial organization of immune evasion mechanisms, these technologies may enable the precise matching of targeted agents, immunotherapies, and conventional chemotherapies to individual patients (23). Furthermore, the temporal monitoring of tumor evolution through integrated approaches may facilitate the implementation of adaptive therapeutic strategies, where treatment regimens are dynamically adjusted based on the emergence of resistance clones, the reconfiguration of the tumor microenvironment, and the evolution of spatially defined molecular signatures. While this paradigm shift from static treatment assignment to dynamic, response-adaptive therapy remains largely conceptual at present, preliminary evidence suggests it holds potential to improve clinical outcomes. Prospective clinical trials are needed to establish the efficacy and feasibility of such approaches (10).

## 6 Challenges and barriers to clinical implementation

Despite the transformative potential of integrating single-cell and spatial multi-omics for precision oncology, several formidable challenges impede the routine clinical implementation of these advanced technologies. These barriers span technical limitations, computational complexities, regulatory and ethical considerations, and economic constraints that collectively determine the feasibility and sustainability of translating research discoveries into clinical practice.

### 6.1 Technical challenges: standardization, reproducibility, and quality control

The clinical adoption of single-cell and spatial multi-omics technologies is critically dependent on the establishment of robust standardization protocols and rigorous quality control measures. Technical variability arising from differences in sample processing, library preparation, sequencing platforms, and data normalization methods continues to pose significant challenges for cross-study comparisons and clinical validation (8). The inherently low input material in single-cell assays renders these technologies particularly susceptible to batch effects, amplification biases, and dropout events that can obscure biologically meaningful signals. Furthermore, the preservation of spatial integrity during tissue preparation, the optimization of antibody staining for spatial proteomics, and the standardization of image analysis pipelines represent ongoing technical hurdles that require systematic resolution before these technologies can be reliably deployed in clinical laboratories (9). The development of reference materials, external quality assessment programs, and consensus guidelines for data acquisition and processing is essential to ensure the reproducibility and clinical validity of multi-omics findings.

### 6.2 Computational challenges: data integration, storage, and analysis pipelines

The computational demands of integrating single-cell and spatial multi-omics datasets represent a substantial barrier to clinical translation. The generation of these technologies produces massive, highdimensional datasets that require sophisticated bioinformatics infrastructure for storage, processing, analysis, and interpretation (4). The integration of heterogeneous data modalities, including transcriptomic, epigenomic, proteomic, and spatial information, necessitates the development of robust computational frameworks capable of handling data heterogeneity, missing values, and technical noise. The harmonization of datasets generated across different platforms and laboratories remains particularly challenging, as batch effects and platform-specific biases can confound biological signals and compromise the generalizability of findings. Furthermore, the deployment of machine learning and artificial intelligence models in clinical settings requires careful validation, interpretability, and continuous monitoring to ensure reliable performance across diverse patient populations (12).

### 6.3 Regulatory and ethical considerations

Regulatory frameworks for the approval and oversight of multi-omics-based diagnostic tests remain in their infancy, with limited guidance on the validation requirements, analytical performance standards, and clinical utility thresholds for spatial biomarkers (5). The generation of detailed molecular profiles raises significant privacy concerns, as these datasets contain sensitive genetic information that could potentially be re-identified or misused. Furthermore, the incidental discovery of germline variants and secondary findings poses ethical dilemmas regarding disclosure, counseling, and informed consent. The equitable access to these advanced technologies is another critical concern, as the high costs of implementation may exacerbate existing disparities in cancer care and limit the benefits of precision oncology to privileged populations.

### 6.4 Cost-effectiveness and scalability

The substantial costs associated with instrumentation, reagents, computational infrastructure, and specialized personnel present significant barriers to implementation, particularly in resource-limited settings and smaller healthcare institutions (8). The current per-sample costs of spatial transcriptomics and single-cell multi-omics remain prohibitively high for routine clinical use, and the scalability of these technologies to accommodate the high-throughput demands of clinical diagnostics remains uncertain. Demonstrating the cost-effectiveness of these approaches through rigorous health economic evaluations, particularly in terms of improved patient outcomes, reduced healthcare utilization, and optimized therapeutic selection, will be essential for securing reimbursement and institutional investment. The development of streamlined workflows, automated analysis pipelines, and scalable computational solutions will be critical for reducing costs and facilitating the broad dissemination of these transformative technologies.

## 7 Future directions and conclusions

### 7.1 Emerging technologies and their potential impact

The next generation of single-cell and spatial multi-omics technologies may further advance precision oncology through enhanced resolution, multiplexing capacity, and clinical accessibility. Emerging platforms combining long-read sequencing with spatial transcriptomics could enable the comprehensive characterization of structural variants and full-length transcript isoforms within their native tissue context (13). Furthermore, the integration of these technologies with CRISPR-based lineage tracing and functional perturbations could enable causal dissection of tumor evolution and therapeutic resistance mechanisms. Artificial intelligence-guided biomimetic nanoplatforms, while still in preclinical development, represent an emerging frontier that builds upon the molecular insights derived from integrated single-cell and spatial profiling to enable targeted therapeutic intervention (15).

### 7.2 Integration with clinical workflows and electronic health records

The successful translation of single-cell and spatial multi-omics into clinical practice will require seamless integration with existing healthcare infrastructure. The development of user-friendly computational platforms that interface with electronic health records may facilitate the interpretation of complex spatial and single-cell molecular data by clinicians and support real-time clinical decision-making (15). Standardized data formats, interoperable software solutions, and automated reporting pipelines are likely to be essential for embedding these technologies into routine oncology workflows.

### 7.3 Toward personalized precision oncology

Achieving truly personalized precision oncology will require rigorous prospective validation, standardization of protocols, and demonstration of clinical utility and cost-effectiveness in real-world settings. The ultimate vision of integrating single-cell and spatial multi-omics is the realization of personalized precision oncology, where treatment decisions are dynamically guided by comprehensive, spatially resolved molecular profiles of individual tumors. This paradigm shift could enable the early detection of resistance, the rational design of combination therapies, and the implementation of adaptive treatment strategies that evolve with the tumor (23).

### 7.4 Concluding remarks

The integration of single-cell and spatial multi-omics represents a transformative approach to understanding and treating cancer. While significant challenges remain, the convergence of technological innovation, computational advances, and clinical translation efforts holds immense promise for improving patient outcomes. The proposed conceptual workflow provides a roadmap for translating these powerful technologies from research discovery to clinical practice. Realizing this potential will depend on continued collaboration between researchers, clinicians, regulatory bodies, and industry partners to overcome the technical, computational, and economic barriers that currently limit widespread adoption.

## References

[1] Inayatullah M Dwivedi AK Tiwari VK . Advances in single-cell omics: Transformative applications in basic and clinical research. Curr Opin Cell Biol. (2025) 95:102548. doi: 10.1016/j.ceb.2025.102548 40517461

[2] Pan D Jia D . Application of single-cell multi-omics in dissecting cancer cell plasticity and tumor heterogeneity. Front Mol Biosci. (2021) 8:757024. doi: 10.3389/fmolb.2021.757024 34722635 PMC8554142

[3] Du Y Ding X Ye Y . The spatial multi-omics revolution in cancer therapy: Precision redefined. Cell Rep Med. (2024) 5:101740. doi: 10.1016/j.xcrm.2024.101740 39293393 PMC11525011

[4] Lim J Park C Kim M Kim H Kim J Lee D-S . Advances in single-cell omics and multiomics for high-resolution molecular profiling. Exp amp; Mol Med. (2024) 56:515–26. doi: 10.1038/s12276-024-01186-2 38443594 PMC10984936

[5] Qureshi Z Altaf F Khanzada M Safi A Asghar Z Warraich D . Liquid biopsies for early detection and monitoring of cancer: advances, challenges, and future directions. Ann Med amp; Surg. (2025) 87:3244–53. doi: 10.1097/ms9.0000000000002776 40486554 PMC12140778

[6] Li Y Ma L Wu D Chen G . Advances in bulk and single-cell multi-omics approaches for systems biology and precision medicine. Briefings Bioinf. (2021) 22(5):bbab024. doi: 10.1093/bib/bbab024 33778867

[7] Wu X Yang X Dai Y Zhao Z Zhu J Guo H . Single-cell sequencing to multi-omics: technologies and applications. biomark Res. (2024) 12(1):110. doi: 10.1186/s40364-024-00643-4 39334490 PMC11438019

[8] Carneiro A Aranda Palomer M Esteves M Rodrigues C Fernandes JM Oliveira F . Advanced microfluidics for single cell-based cancer research. Adv Sci. (2025) 12(41):e72228. doi: 10.1002/advs.202500975 40641450 PMC12591218

[9] Cen X Huang X Deng E Gong X Tan N Ye J . Single-cell and spatial omics: Methods and applications. MedComm. (2026) 7(4):70713. doi: 10.1002/mco2.70713 41953641 PMC13053676

[10] Tang X Berger MF Solit DB . Precision oncology: current and future platforms for treatment selection. Trends Cancer. (2024) 10:781–91. doi: 10.1016/j.trecan.2024.06.009 39030146

[11] Kim YJ Takahashi K . Emerging technologies of single-cell multi-omics. Haematologica. (2025) 110(6):1269–77. doi: 10.3324/haematol.2022.282557 39911109 PMC12130758

[12] Ma J Dong C He A Xiong H . Single-cell multiomics: a new frontier in drug research and development. Front Drug Discov. (2024) 4. doi: 10.3389/fddsv.2024.1474331

[13] Guzman G Rodriguez A . Long-read sequencing for cancer liquid biopsy: advancing precision oncology. Front Oncol. (2026) 16. doi: 10.3389/fonc.2026.1811012 42358548 PMC13290525

[14] Liu F Beck S Yang L Luo H Zhang K . Advancing ai for multi-omics and clinical data integration in basic and translational cancer research. Nat Rev Cancer. (2026) 26:497–512. doi: 10.1038/s41568-026-00922-2 42014628

[15] Li J Liu W Mu Y Wang X Zhang H Tang K . Artificial intelligence-assisted spatial omics-based biomimetic nanoplatform for intelligent and precise intervention in the immunosuppressive core region of ovarian cancer. NPJ Precis Oncol. (2026) 10(1):01345-w. doi: 10.1038/s41698-026-01345-w 41814059 PMC13111681

[16] Huang X Huang S Reina C Šabanović B Roberto M Aicher A . Single-cell multi-omics and machine learning for dissecting stemness in cancer. Briefings Bioinf. (2025) 26(5):bbaf566. doi: 10.1093/bib/bbaf566 41159730 PMC12570030

[17] Li Y Qiu H Zhao Z Qi F Cai P . Single-cell technologies and spatial transcriptomics: decoding immune low - response states in endometrial cancer. Front Immunol. (2025) 16. doi: 10.3389/fimmu.2025.1636483 40672944 PMC12263668

[18] Barjij I Lamsyah O Kdadri S Lkhoyaali S Najem S Naciri S . Spatial and temporal intratumoral heterogeneity in breast cancer: a systematic and conceptual review of single-cell and spatial omics studies. BMC Cancer. (2026) 26:15928-0. doi: 10.1186/s12885-026-15928-0 41942920 PMC13188440

[19] Le J Dian Y Zhao D Guo Z Luo Z Chen X . Single-cell multi-omics in cancer immunotherapy: from tumor heterogeneity to personalized precision treatment. Mol Cancer. (2025) 24:(1):221. doi: 10.1186/s12943-025-02426-3 40855431 PMC12376342

[20] Veo B Wang D DeSisto J Pierce A Brunt B Bompada PC . Single-cell multi-omics identifies metabolism-linked epigenetic reprogramming as a driver of therapy-resistant medulloblastoma. Nat Commun. (2025) 16(1):10470. doi: 10.1038/s41467-025-65466-9 41290646 PMC12647721

[21] Nomura M Raviram R Schiffman JS Bussema L Lu V Wheeler N . Longitudinal changes in dna methylation in idh-mutant glioma fuel disease progression through altered cell state differentiation. Nat Genet. (2026) 58(7):1651–60. doi: 10.1038/s41588-026-02642-7 42332268 PMC13364636

[22] Tang N Li J Gu A Li M Liu Y . Single-cell multi-omics in biliary tract cancers: decoding heterogeneity, microenvironment, and treatment strategies. Mol BioMed. (2025) 6(1):82. doi: 10.1186/s43556-025-00330-2 41091402 PMC12528628

[23] He J Xue W Li Y . Tumor microenvironment-responsive nanomedicine: Monitoring and modulating the tumor microenvironment for precision cancer therapy. Int J Nanomed. (2026) 21:1–38. doi: 10.2147/ijn.s560983 41884284 PMC13011976

[24] Weng S Cain L Comben J Zhang Y Semple T Alaei S . Recurrent intratumour heterogeneity is a hallmark of metastatic prostate cancer. Nat Commun. (2026) 17(1):7727. doi: 10.1038/s41467-026-74334-z 42321191 PMC13434311

[25] Nirob SR Morol MK Rahman D Hui LT Nandi D Rahman M . Early multi-cancer detection using liquid biopsy: emerging biomarkers and clinical strategies. Front Oncol. (2026) 16. doi: 10.3389/fonc.2026.1877911 42490792 PMC13375447

